# Technologies That Assess the Location of Physical Activity and Sedentary Behavior: A Systematic Review

**DOI:** 10.2196/jmir.4761

**Published:** 2015-08-05

**Authors:** Adam Loveday, Lauren B Sherar, James P Sanders, Paul W Sanderson, Dale W Esliger

**Affiliations:** ^1^ National Centre for Sport and Exercise Medicine School of Sport, Exercise and Health Sciences Loughborough University Loughborough United Kingdom; ^2^ National Institute for Health Research (NIHR) Leicester-Loughborough Diet, Lifestyle and Physical Activity Bio-medical Research Unit Leicester United Kingdom

**Keywords:** wearable camera, global positioning system, real-time locating system, sitting, context

## Abstract

**Background:**

The location in which physical activity and sedentary behavior are performed can provide valuable behavioral information, both in isolation and synergistically with other areas of physical activity and sedentary behavior research. Global positioning systems (GPS) have been used in physical activity research to identify outdoor location; however, while GPS can receive signals in certain indoor environments, it is not able to provide room- or subroom-level location. On average, adults spend a high proportion of their time indoors. A measure of indoor location would, therefore, provide valuable behavioral information.

**Objective:**

This systematic review sought to identify and critique technology which has been or could be used to assess the location of physical activity and sedentary behavior.

**Methods:**

To identify published research papers, four electronic databases were searched using key terms built around behavior, technology, and location. To be eligible for inclusion, papers were required to be published in English and describe a wearable or portable technology or device capable of measuring location. Searches were performed up to February 4, 2015. This was supplemented by backward and forward reference searching. In an attempt to include novel devices which may not yet have made their way into the published research, searches were also performed using three Internet search engines. Specialized software was used to download search results and thus mitigate the potential pitfalls of changing search algorithms.

**Results:**

A total of 188 research papers met the inclusion criteria. Global positioning systems were the most widely used location technology in the published research, followed by wearable cameras, and radio-frequency identification. Internet search engines identified 81 global positioning systems, 35 real-time locating systems, and 21 wearable cameras. Real-time locating systems determine the indoor location of a wearable tag via the known location of reference nodes. Although the type of reference node and location determination method varies between manufacturers, Wi-Fi appears to be the most popular method.

**Conclusions:**

The addition of location information to existing measures of physical activity and sedentary behavior will provide important behavioral information.

## Introduction

Physical activity has a long-established relationship with several chronic conditions including diabetes, heart disease, and certain forms of cancer [1]. Recent evidence suggests that sedentary behavior carries deleterious effects on health outcomes independent of moderate-to-vigorous physical activity (MVPA) in young people [2] and adults [3], although this is not a uniform finding [4]. Sedentary behaviors are defined as any waking activity with an energy expenditure of ≤1.5 metabolic equivalents (METs) while in a sitting or reclining position [5]. A paradigm shift is underway toward an increasing appreciation of the importance of reducing sedentary time alongside increasing physical activity [6].

Within the behavioral epidemiology framework [7], the location of a behavior may influence the correlates of the behavior and the intervention strategies needed to change behavior. Discerning the varying contribution of multiple locations to physical activity and sedentary time will allow researchers to target interventions to locations which are associated with the lowest levels of physical activity or highest levels of sedentary time. Understanding the contribution of multiple locations to health behaviors first requires the accurate measurement of location, as suggested by the behavioral epidemiology framework [7,8].

Sedentary behavior and physical activity differ in the domains and locations in which they are likely to occur. Sedentary time is likely, though not exclusively, to occur indoors at the home, at work or school, or in leisure pursuits such as eating a meal or going to the cinema. Conversely, MVPA may occur through active transport, housework, or purposeful exercise. This can be illustrated through the close link between adults, on average, spending approximately 90% of time indoors [9,10] and approximately 60% of time in sedentary activities [11]. The large proportion of time spent indoors and the increasing research focus on sedentary behavior suggest that an accurate measure of where behavior occurs indoors would be particularly valuable.

Determining where physical activity and sedentary time are performed will provide valuable information in isolation; however, it can also act in a synergistic manner. For example, much recent effort has focused on the use of complex pattern recognition techniques to determine the mode or type of activity being performed from raw acceleration data. Depending on the classification method used, classification accuracies between 50% and 90% have been achieved [12]. Given the probabilistic nature of these activity classification methods, the inclusion of location-based data into the current algorithms may provide greater levels of accuracy. For instance, the likelihood of stair climbing is greatly increased if an individual is near a staircase. Similarly, context-sensitive questioning via ecological momentary assessment (EMA) [13] can be enhanced by using location to trigger desirable questions in place of time-based cues.

Furthermore, measurement of indoor location could benefit research into the correlates of physical activity or sedentary behavior. For example, the presence of a television set in a child’s bedroom may be a correlate of higher screen time [14]; however, this may be a stronger correlate for those who spend more time in their bedrooms. Establishing how much time a child spends in their bedroom via objective indoor location could, therefore, fully elucidate the strength of this correlate. Thus, the accurate measurement of location could greatly enhance several areas within physical activity and sedentary behavior research, both in and of itself and as an adjunct to other research areas.

Individuals may be able to accurately report the broad location of their physical activity and sedentary behavior [15]; however, self-report location instruments are unable to provide detailed and temporally patterned location information. Objective monitoring could, therefore, provide a more robust means to measure the location of physical activity and sedentary behavior. To date, time indoors has been inferred through the lack of a global positioning system (GPS) signal [16] or through the use of a light (lux) sensor incorporated into activity monitors [17]. However, these methods are only able to differentiate indoor from outdoor and do not provide room- or subroom-level location. Alongside measures of outdoor location, there is, therefore, a need for measures of room- and subroom-level indoor locations, which are feasible for use in this field of research. This review aims to provide an overview of devices and technology currently used, or that could potentially be used, to assess the indoor or outdoor location of physical activity and/or sedentary behavior.

## Methods

### Search Strategy

Search strategies to identify potentially relevant articles were built around three key groups of keywords: behavior, measurement, and context. Key terms were as follows: sedentary lifestyle, sedentary lifestyles, sedentary behav*, screen time, seden*, sitting time, motor activity, motor activities, physical activity, or activities of daily living; measur*, assess*, patterns, monitor, or sensor; and context*, setting, location, mode, domains, or environment. Scopus, Web of Science, PubMed, Institute of Electrical and Electronics Engineers (IEEE), and OpenGrey were searched using the key terms up to February 4, 2015. Subsequently, forward and backward searching of included articles (ie, references and articles citing the included article) was conducted to identify any further eligible articles. In addition, manual searches of personal files were conducted.

### Inclusion and Exclusion Criteria

To be included in this review, studies were required to meet the following criteria: (1) be published in the English language, (2) either describe a tool used to measure the location of physical activity and/or sedentary behavior or provide sufficient information to discern whether the instrument could be modified to measure location, and (3) be a portable/wearable tool. Technologies were required to be portable or wearable to ensure that the technology is always with the participant and that the scope of the review was not so broad as to be unmanageable by including nonwearable technologies (eg, closed-circuit television [CCTV]). A minimum of one part of the measurement system, not the whole system, was required to be wearable/portable for inclusion. For example, GPS systems consist of a wearable unit and orbiting satellites (ie, one part of the system is wearable but the whole system also consists of unwearable components). Wearable technologies is also an area which is experiencing rapid growth in the consumer sector, as technology increasingly becomes smaller, more powerful, and multi-purpose. Wearable technologies, therefore, give this review a contemporary positioning. No date restriction was placed on search results. Studies erroneously defining sedentary behavior as the absence of sufficient physical activity rather than activities undertaken in a sitting or reclined position [5], were treated as physical activity studies.

### Identification of Relevant Studies

Titles and then abstracts of identified articles were screened to determine eligibility based on the above inclusion criteria. Titles and abstracts which did not meet the inclusion criteria were excluded. Following this, the full text of any potentially relevant article was obtained for full reading to determine conformity to the inclusion criteria. A subsample of potentially relevant articles retrieved for full-paper screening were extracted by a second author (JPS) to determine interrater agreement. If any discrepancies arose, these were resolved by discussion between authors. Interrater agreement was high (Cohen’s kappa = .81).

### Data Extraction and Synthesis

Data of eligible papers was extracted via standardized forms developed for this review. All available information was extracted. Identified devices which assessed where physical activity and sedentary behavior occur were tabulated to highlight the available literature in this research area and to showcase the array of measurement technologies.

### Internet Search Engines

To ensure that the widest possible range of devices were included, systematic searches of Internet search engines were performed for devices and technologies that are able to measure location but may not have made their way into the published research to date. This was necessary due to the relatively slow pace of research and publication compared to the pace of technological advance (ie, new research papers may use old technology which has been surpassed by newer models). Google, Bing, and Yahoo were searched using the following key terms: RTLS (real-time locating system), GPS tracking device, RFID (radio-frequency identification) tracking, wearable camera, wearable GPS, and wearable RFID. These search terms were chosen based on the results of the academic literature searches. Specialized software was used to export the first 300 results of each search to Microsoft Excel. This ensured that the results were unaffected by the changing algorithms of search engines. Searches were completed on February 4, 2015. The retrieved website addresses were screened to determine eligibility. Only manufacturer websites were included to ensure the accuracy of the information. All other websites, including blogs and consumer review websites, were excluded. Eligible websites were then browsed for location monitoring devices. Only devices and full integrated systems which are ready to use (ie, not bespoke) were included in an attempt to address the practicalities of deployment to assess where physical activity and sedentary time occur. The specifications of these devices were then extracted using standardized forms developed for this review. If available, specifications were obtained from device manuals. If device manuals were not available, any specifications shown on the website regarding the device were extracted. Only available information was extracted (ie, gaps in tables indicate a lack of available information). By note of caution, readers should be mindful that device characteristics, as supplied by manufacturers, are often generated under ideal conditions. Real-world pilot-testing with participants may, therefore, be required to establish real-world device characteristics.

## Results

**Figure 1:**
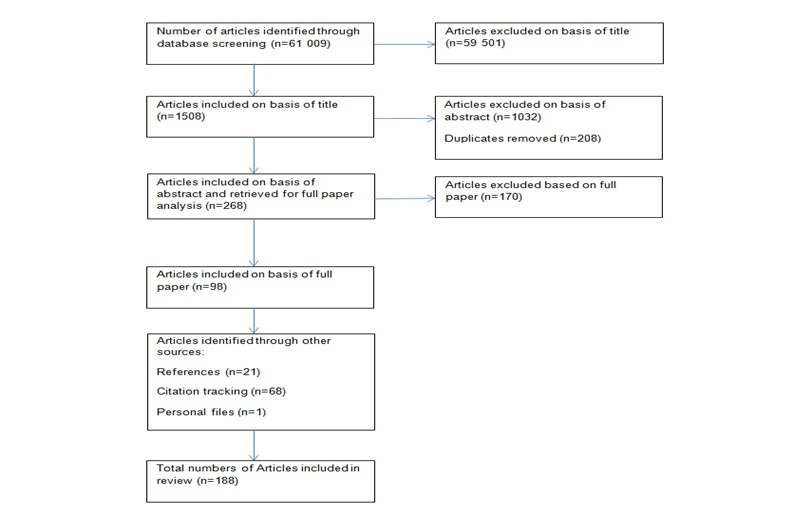
Flowchart of study selection process.

The number of research papers included and excluded at each stage of the systematic review process is shown in Figure 1. This review began with 61,009 potentially eligible papers, eventually resulting in the full inclusion of 98 papers. A further 90 papers were then identified through reference searching, citation tracking, and the searching of personal files.

**Figure 2:**
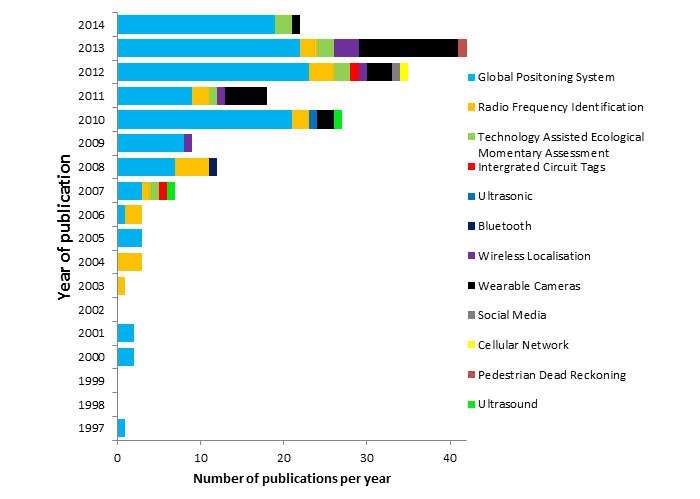
Number of studies published each year covering different types of technology. A total of 12 kinds of technology were found during the course of this review.

A breakdown by year and technology is depicted in Figure 2. This review found 12 types of technology capable of assessing where physical activity and sedentary behavior occur. GPS was the most widely used location monitoring technology, comprising 119 (63.3%) [16,18-134] of the total 188 papers. Wearable cameras and RFID were the second- and third-most popular forms of location technology, contributing 23 (12.2%) [18,19,135-156] and 20 (10.6%) [157-177] studies, respectively, out of 188. The remaining 9 technologies each contributed a small number of studies (8 [4.3%] or less) to the total sample [178-200]. GPS has the longest history of use, initially being used within sports science in 1997. Conversely, wearable cameras and Wi-Fi-based localization technologies appear to be the most recent debut within research.

Selective details of devices used within research are shown in Table 1 (wearable cameras), Table 2 (GPS), and Table 3 (other). A complete version of Table 2 is available as Multimedia Appendix 1.

Tables 4-6 show selective characteristics of the results of the Internet search engine searches for wearable cameras, RTLS, and GPS, respectively. Complete versions of Tables 5 and 6 are available as Multimedia Appendices 2 and 3. These searches found 21 wearable cameras [201-214], 78 RTLS tags from 35 companies [215-249], and 81 GPS devices [250-286]. GPS devices were marketed for a variety of purposes, including the tracking of children by parents, elder monitoring to limit wandering, and the tracking of young drivers. RTLS companies positioned their products as suitable for asset management applications in warehouses and, to a lesser extent, equipment and patient tracking in health care settings. Wearable cameras were targeted toward extreme sports, life logging, and law enforcement applications.

**Table 1:** Summary of wearable camera systems used in published research to date.

Man^a^	Model	I/O^b^	BL^c^, h	CR^d^, mp	Dim^e^, cm	Weight, kg or g	Wear site	SF^f^, FPS^g^	Refs^h^, notes
Natural- Point, Inc	OptiTrack- Prime 17W	Indoor	N/A^i^	1.7	12.6x12.6 x11.0	1.32 kg	N/A	30-360	[135]
Vicon	Motion capture system	Both (most indoor)	N/A	≤16	N/A	N/A	N/A	≤1000	[136,137]
Prototype	eButton	Both	~10	N/A	6.2 diameter	42 g	Pin onto shirt	10	[138-140] Not yet commer-cialized
Prototype	Prototype Wrist- Sense	Both	~7	N/A	N/A	N/A	Wrist	6	[141,142]
Microsoft	SenseCam (Vicon Revue)	Both	≤16	N/A	N/A	N/A	Lanyard around neck	Change in sensor readings	[18,19,143-155] Available as Auto- grapher
Looxcie	Looxcie 2	Both	1-4	N/A	2.31x1.70 x8.46	22 g	N/A	15/30	[156]

^a^Man: manufacturer

^b^I/O: Indoor/outdoor

^c^BL: battery life

^d^CR: camera resolution

^e^Dim: dimensions

^f^SF: sampling frequency

^g^FPS: frames per second

^h^Refs: references

^i^N/A: not applicable

**Table 2:** Summary of Global positioning systems used to date in published research (see Multimedia Appendix 1 for the full version of this table).

Man^a^	Model	Battery life, h, days, or weeks	Dim^b^, cm or mm	Weight, g	Wear site	Cold start time, s or Hz	Storage, points, MB, or, GB	References
Garmin	Foretrex 201	15 h	8.4x4.3x 1.8 cm	78	Wrist	45 s	10,000 points	[16,20-31]
Garmin	Forerunner 305	Typically 10 h	5.3x 6.8x 1.7 cm	77	Wrist	45 s	N/A^c^	[32-35]
Garmin	Forerunner 205	10 h	53x69x 18 mm	77	Wrist	45 s	72,000 points	[25,30,42-45]
Garmin	60	N/A	N/A	N/A	Pocket of back- pack	0.5 Hz	N/A	[46,47]
Telespial Systems	Trackstick II	16-36 h, 2 days- 1 week in power save	11.4x3.1x 1.9 cm	N/A	N/A	Maximum of 52 s	1 MB	[52,53]
GlobalSat	DG100	20-24 h	N/A	N/A	Waist	5, 15, or 30 s	50,000 points	[25,47,54-59]
GPSports	SPI ELITE	N/A	N/A	N/A	Back harness	1 Hz	N/A	[60-69]
GPSports	SPI PRO	N/A	N/A	N/A	Back harness	5 Hz	N/A	[71-74]
GPSports	SPI 10	N/A	N/A	N/A	Back harness	1 Hz	N/A	[65,66,73,75-77]
Catapult Innovations	MinimaxX	5 h	8.8x5.0x 1.9 cm	67	Back harness		1 GB	[62,73,78-87]
Telespial Systems	Super	4-8 days	N/A	N/A	Waist	5 or 15 s	N/A	[88,89]
Qstarz	BT1000X	42 h	72x47x 20 mm	65	Pouch on belt	35 s, 5 s, or 15 s	400,000 points	[18,19,30,59,90-104,134]
GlobalSat	BT335	25 h	N/A	N/A	Waist	30 s	N/A	[110-114]

^a^Man: manufacturer

^b^Dim: dimensions

^c^N/A: not applicable

**Table 3:** Summary of other measures used in published research to date.

Type of measure	Indoor/outdoor	References
Radio-frequency identification	Indoor	[157-177]
Wireless localization	Indoor	[178-183]
Technology-assisted ecological momentary assessment/experience sampling	Both	[184-191]
Integrated circuit tags	Indoor	[192,193]
Ultrasonic (Bat system)	Indoor	[194]
Cellular networks	Outdoor, but works indoor	[195]
Bluetooth	Indoor	[196]
Social media check-in	Both	[197]
Ultrasound	Indoor	[198,199]
Pedestrian dead reckoning system	Indoor	[200]

**Table 4:** Summary of commercially available wearable cameras unused in research to date.

Manufacturer, reference	Model	Battery life of wearable component, h or min	Dimensions, mm, in, or cm	Weight, g or oz	Wear site
Autographer [201]	N/A^a^	10 h	37.40x90.00x 22.93 mm	58 g	Clip or lanyard
**Narrative (formally Memoto) [202]**					
	Clip	N/A	36x36x9 mm	20 g	Clip or lanyard
	Clip 2 (released spring 2015)	N/A	N/A	N/A	N/A
**MeCam [203]**					
	Classic	80 min continuous	1.75x0.50 in	1 oz	Clip or necklace
	MeCam HD	60-120 min	2x2 in	2.5 oz	N/A
**uCorder [204]**					
	Pockito IRDC260-R	≤75 min	2.50x1.25x0.50 in	N/A	N/A
	Pockito IRDC260-B	≤75 min	2.50x1.25x0.50 in	N/A	N/A
	Pockito IRDC150	≤2 h	1.1x0.6x3.5 in	N/A	N/A
	Pockito IRDC250	≤2 h	1.1x0.6x3.5 in	N/A	N/A
ParaShoot [205]	2.1	N/A	45x4x15 mm	1.5 oz	Clip
Spy Emporium [206]	Spy hidden camera glasses	1-2 h	160x40x40 mm	N/A	Glasses/ on face
**VIEVU [207]**					
	VIEVU 2	2.5 h recording, 1.5 h streaming	1.90x1.90x0.75 in	2.4 oz	Clip
	LE3	≤5 h	3.00x2.10x0.85 in	2.8 oz	Clip
**Panasonic [208]**					
	WV-TW310L	5 h continuous	45x75x41 mm	210 g	
	WV-TW310S	5 h continuous	45x75x41 mm	160 g	
meMINI [209]	N/A	3.5 h	N/A	N/A	Lanyard
Pivothead [210]	N/A	N/A	N/A	N/A	Glasses/ on face
Nixie [211]	N/A	N/A	N/A	N/A	Wrist (detaches to become camera)
CA7CH [212]	Lightbox	N/A	38x38x10 mm	30 g	Clip
ELMO USA [213]	QBIC-MSI	2 h	2.14x2.40x1.57 in	95 g	Lanyard
Vidcie [214]	Lookout QUB	1 h (8 h with battery pack)	4.8x4.8x1.5 cm	37 g	Clip

^a^N/A: not applicable

**Table 5:** Summary of commercially available real-time locating systems unused in research to date (see Multimedia Appendix 2 for the full version of this table).

Manufacturer, reference	Model	Infrastructure/ method	Dimensions, mm, in, or cm	Accuracy, m, cm, or ft
**Ekahau [215]**				
	A4	Wi-Fi, RSSI^a^and triangulation	45x55x19 mm	1 m
	B4	Wi-Fi, RSSI and triangulation	60.0x90.0x8.5 mm	1 m
	W4	Wi-Fi, RSSI and triangulation	51.5x50.0x17.5 mm	1 m
**Ubisense [216]**				
	Series 7000 industrial	UWB^b^, TOA^c^, AOA^d^	71x64x47 mm	15 cm
	Series 7000 compact	UWB, TOA, AOA	38.0x39.0x16.5 mm	15 cm
	Series 7000 slim tag	UWB, TOA, AOA	83x42x11 mm	15 cm
	Series 700 intrinsically safe tag	UWB, TOA, AOA	38.0x39.0x25.5 mm	15 cm
	Series 9000 compact tag	UWB, TOA, AOA	38.0x39.0x16.5 mm	15 cm
**Zebra [218]**				
	WhereTag IV	Wi-Fi, TDOA^e^	43.7x66.0x21.3 mm	2 m
	WhereTag III	Wi-Fi, TDOA	21x66x44 mm	
Sonitor [222]	Whole system	Wi-Fi, ultrasound, RSSI	N/A^f^	1 ft
Secure Care [225]	ENVisionIT	Wi-Fi	N/A	30 cm
Mojix [226]	eLocation	Passive RFID^g^	N/A	Within 1 m
TempSys [228]	Fetch System	RF^h^and ultrasound, TDOA	N/A	0.5 m
**Awarepoint [229]**				
	Asset tags	ZigBee	1.8x1.3x0.5 in	Up to bay level
	Wearable tag	ZigBee	1.8x1.3x0.5 in	
Nebusens [232]	Sirius Quantum	ZigBee	22.00x32.72x5.00 mm	1 m
Essensium [233]	Mobile nodes	Wide over narrowband RF, TWR^i^, TOF^j^	19.8x8.8 cm	Typically 50 cm
**PLUS Location [234]**				
	R1 badge tag	UWB, TDOA	38.0x78.0x9.6 mm	<1 m
	R2 tags	UWB, TDOA	87x42x10 mm	<1 m
Purelink [239]	Personnel tracking tag	RFID	85x54x4 mm	2 m
**Sanitag [240]**				
	Staff tag	RF, RSSI, TOF	90x61x5 mm	2.5 m
	Patient tag	RF, RSSI, TOF	43x36x10 mm	2.5 m
OpenRTLS [242]	Tag	UWB, TDOA, TWR	66x44x17 mm	10 cm

^a^RSSI: received signal strength indicator

^b^UWB: ultra wide band

^c^TOA: time of arrival

^d^AOA: angle of arrival

^e^TDOA: time difference of arrival

^f^N/A: not applicable

^g^RFID: radio-frequency identification

^h^RF: radio frequency

^i^TWR: two-way ranging

^j^TOF: time of flight

**Table 6:** Summary of commercially available global positioning systems unused in research to date (see Multimedia Appendix 3 for the full version of this table).

Manufacturer	Model	Battery life of wearable component, days, weeks, months, or h	Dimensions, in, cm, or mm
**Trackstick [250]**			
	Trackstick mini	3-14 days	3.50x1.50x0.38 in
	Trackstick II	16 h-2 days (AAA)	4.50x1.25x0.75 in
	Super Trackstick	3 days-3 weeks (AAA)	4.50x1.25x0.75 in
Trackershop-UK [251]	Pro-pod5	14-15 days	6.35x4.00x2.50 cm
**Gotek7 [252]**			
	Prime 1.0	10 days normal; ≤12 months with 1 update per day	N/A^a^
	Prime 2.0	15 days normal; ≤14 months (1 per day)	65x42x25 mm
**Trackinapack [256]**			
	Advanced	≤10 days	2.63x1.38x0.79 in
	Advanced plus	≤15 days	2.50x1.50x0.79 in
**TracLogik [259]**			
	Guardian GPS	100-220 hours	67.8x37.0x20.0 mm
	Guardian pro GPS	2-14 days	62.5x40.0x25.0 mm
	Covert 2000	10-15 days	61x34x31 mm
Loc8tor [261]	N/A	≤9 months in power save; 3-14 days normally	68x36x20 mm
**LandAirSea [269]**			
	Silvercloud realtime GPS tracker	5-6 days at 2 h per day	3.90x2.26x0.90 in
	Tracking key pro	2 weeks (4 h), 4 weeks (2 h), 6 weeks (1 h per day)	3.01x1.95x1.40 in
**GTX Corp [273]**			
	Prime AT	≤16 days	67x37x20 mm
	Smart sole	2-3 days	Depends on show size
Nike [276]	Sportwatch GPS	8 h with average use	1.5x10.1x0.6 in
**Garmin [277]**			
	Forerunner 620	6 weeks (watch) 10 h (training)	45.0x45.0x12.5 mm
	Forerunner 220	6 weeks (watch) 10 h (training)	45.0x45.0x12.5 mm
	Tactix	50 h (5 weeks in watch mode)	49x49x17 mm
	Fenix 2	20 h ( 5 weeks in watch mode)	49x49x17 mm
Trax [280]	Trax	1 day	38x55x10 mm
**Personal GPS Trackers [282]**			
	Personal GPS Tracker	≤7 days	65x40x18 mm
	Mini GPS Tracker	2-4 days	58x22x11 mm

^a^N/A: not applicable

## Discussion

### Principal Findings

This systematic review sought to identify tools which have been used, or could be modified for use, to assess where physical activity and sedentary behaviors occur. This review identified 188 research papers which used 12 different types of technology. The most widely used technology was GPS with 119 publications [16,18-134], followed by wearable cameras and RFID with 23 [18,19,135-156] and 20 [157-177] publications, respectively. The remaining 9 types of technology each contributed a small number of studies to the total sample [178-200]. However, it should be noted that a number of these were bespoke or prototype systems; this is particularly true of RFID, integrated circuit (IC) tag systems, and various communication protocols for wireless localization.

Systematic grey searches identified 21 wearable cameras [201-214], 78 RTLS tags [215-249], and 81 GPS devices [250-286]. By only including devices which are "ready to use," we sought to address the practicalities of deployment and limit the inclusion of bespoke technologies. Combined with the devices used within research papers to date, we identified a total of 263 devices. The history, principles of use, and the applications for GPS, RTLS, and wearable cameras will now be discussed in greater detail.

### Global Positioning System

Originally developed by the United States Department of Defense, the GPS system consists of 24 satellites orbiting Earth. These satellites transmit signals to GPS receivers and are able to determine the location, direction, and speed of the receiver based on trilateration between three or more satellites [287]. Due to the original military application of GPS, a deliberate error was embedded into the system to reduce the risk of enemy forces using the system. This deliberate error was removed in the year 2000, thus making the system available to civilian users. The use of GPS has since proliferated into areas such as criminal offender tracking, vehicle tracking, and vehicle navigation. Such has been the widespread adoption of GPS, that the European Union is currently investing substantial amounts of money into its own satellite system to ensure it is not reliant on American satellites. Early GPS devices possessed limited battery life and memory capacity and form factors unsuitable for long periods of wear. Thus GPS devices were first used for sports applications before making their way into health research.

The earliest GPS study in a sporting domain was conducted in 1997 [132]. It was found from this initial evaluation that GPS could be used to assess human locomotion [132]. Following this early study, GPS has been used to assess movement characteristics in sports such as Australian football [66], orienteering [49], hockey [63], and rugby [72]. These studies have generally found GPS to be a suitable measure of movement parameters in sport, such as speed and distance. Physiological measures such as heart rate are often included alongside GPS to provide further data on the demands of a particular sport. These devices are often worn on the back via a custom-made vest and are, therefore, unlikely to be suitable for long-term wear. These sports studies, therefore, provide little insight into the applicability of GPS for assessing free-living physical activity.

The earliest study to use GPS to investigate free-living physical activity was conducted in 2005 [22]. The GPS units were found to provide valid and reliable measures of location when compared to a known geodetic point [22]. Following the validation of these units, a small pilot study examined the feasibility of integrating GPS, geographic information system (GIS), and accelerometer data. It was found that GPS and accelerometer data could be successfully integrated, with GPS data available for 67% of all MVPA time [22]. Accelerometer, GIS, and GPS data have since been successfully integrated in further studies to assess active commuting to school [16] and time spent outdoors after school [20].

In reviewing 24 studies which use GPS in physical activity research [288], GPS data loss was found to be highly correlated with device wear time (r=.81, *P*<.001). Common reasons for data loss include signal dropout, limited battery power, and poor protocol adherence [288]. Due to devices requiring a line of sight to the orbiting satellites, signal dropout can occur when this line of sight is broken. The necessity for GPS devices to have a line of sight to at least three orbiting satellites also results in GPS only receiving signal within certain indoor environments, such as a single-story building with a wooden roof or high-story building with large windows. Even under these circumstances, GPS is unable to determine room- or subroom-level indoor location. Participants are often required to remain stationary outside before commencing a journey to ensure that the GPS device can acquire satellite signal, failure to adhere to this can result in data loss.

While GPS can be used to successfully augment accelerometer measurement of physical activity, several shortcomings need to be addressed. There is currently no established approach to the analysis and interpretation of GPS data [287]. Guidelines and common data analysis programs for the capture and analysis of GPS data, such as the Personal Activity and Location Measurement System (PALMS), are therefore highly useful in standardizing approaches. Due to requiring a clear line of sight to orbiting satellites, GPS is most suitable for assessing outdoor location. However, up to 90% of our time is spent indoors [9,10]. The ability to assess where physical activity and sedentary time occur in an indoor environment would allow the formation of a more comprehensive behavioral profile which incorporates contextual information alongside accelerometry-measured intensity and duration.

### Wireless Localization

Wireless localization technology has been commercialized under the umbrella term real-time locating systems. Used in health care [289] and warehouse environments, RTLS systems are able to assess the location of people or assets within an indoor environment. Many RTLS devices are commercially available (see Table 5 and Multimedia Appendix 2). All of these devices function on the principle of determining the location of a mobile component via the known location of fixed components, though the method of determining location and the type of fixed component vary between manufacturers. Interested readers are referred elsewhere for detailed technological reviews of wireless localization [290-293].

**Figure 3:**
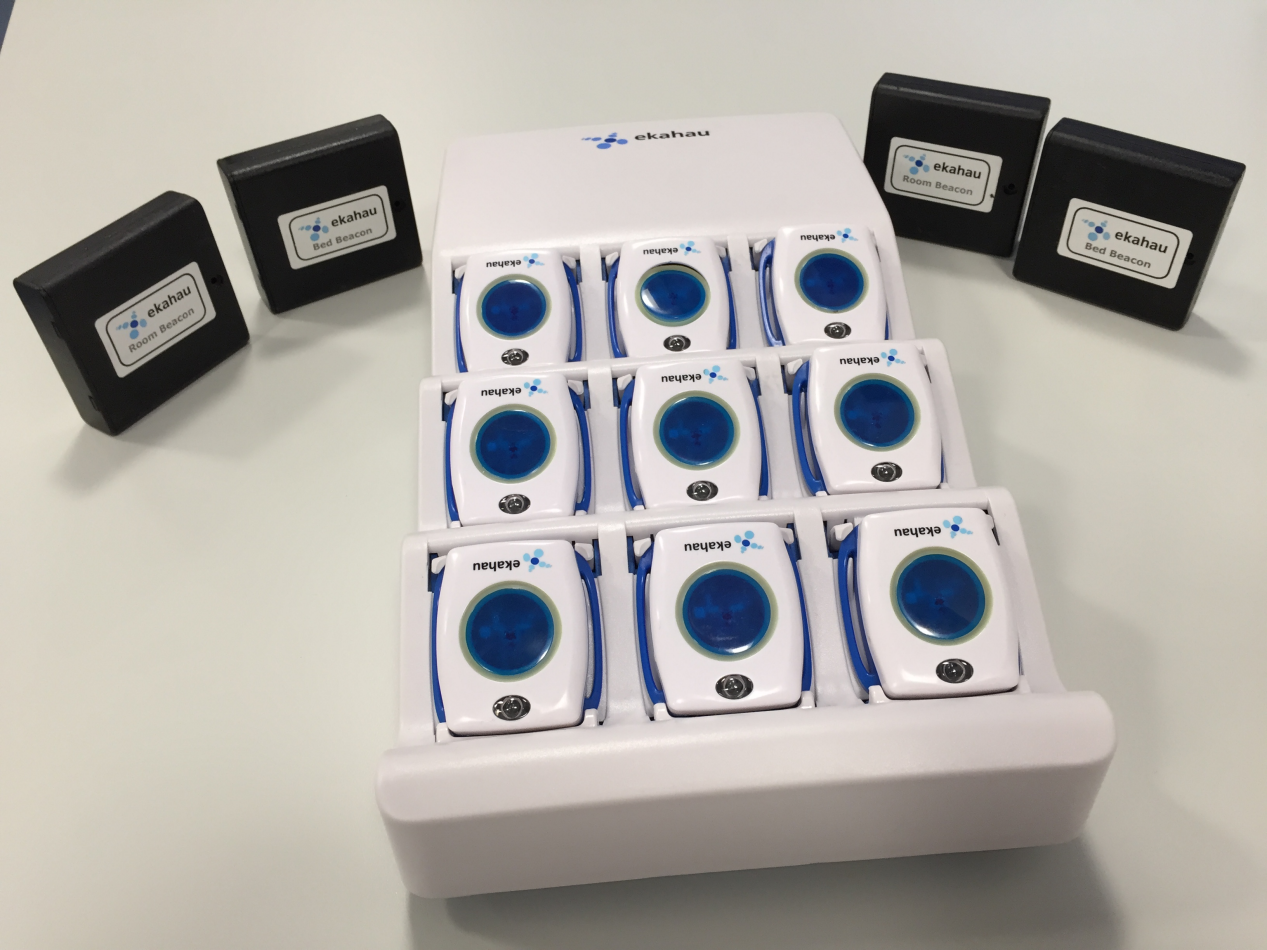
Hardware components of the Ekahau real-time locating system (RTLS) [214]. Wearable T301W white wrist tags are shown in a charging cradle; infrared beacons are shown on either side of the charging cradle.

The fixed components of RTLS systems also vary between RTLS manufacturers. Some manufacturers, such as AeroScout, require the installation of proprietary fixed reference points. Others, such as the Ekahau system, are able to utilize existing Wi-Fi points within buildings as fixed reference points and, therefore, do not require the installation of infrastructure. Several manufacturers also provide infrared (IR) location beacons for increased location accuracy in areas of poor signal strength. Hardware of the Ekahau RTLS system is shown in Figure 3. The location of the mobile component of the RTLS system, worn by an individual or placed on equipment, is then relayed back to software supplied with the RTLS system. This software requires a floor plan of the environment being monitored; the location of the mobile component is then viewed on this floor plan or as an x and y coordinate. RTLS systems, therefore, function in much the same manner as GPS: providing x and y coordinates rather than longitude and latitude. The manufacturers of several RTLS systems suggest that their systems are capable of handling hundreds of mobile tags simultaneously. Manufacturers state that RTLS systems are generally accurate to within 2 to 3 meters.

However, RTLS systems are not without limitations. Due to their predominant use in the tracking of patients and equipment, many RTLS systems are configured for real-time monitoring and require slight modification to generate a log of coordinates for any later integration with other data streams. At present, RTLS systems are not being used in physical activity or sedentary behavior research; therefore, the feasibility of incorporating RTLS data with accelerometry is unknown. The RTLS software requires the manual setting of the scale of the floor plan and, therefore, introduces possible human error into the system.

Despite this, RTLS could potentially be used within physical activity and sedentary behavior research to answer a number of research questions which are currently assessed via self-report methods. For example, RTLS, alongside accelerometry, could provide location information to assess whether youngsters in a daycare center are more likely to be active when they are near equipment such as a sandbox or when they are near other active youngsters. Likewise, if researchers are undertaking a standing desk intervention to reduce sitting time, participants are currently often asked to self-report how much time they spend at their desk. The amount of time the participant spends at their desk may impact any possible reduction in sitting time due to the standing desk. With RTLS, researchers would be able to objectively determine the amount of time their participants were at their standing desk and thus determine the success, or otherwise, of the intervention with greater certainty.

Determining the indoor location of physical activity and sedentary behavior via RTLS may also be an important research finding in itself. For example, within an elderly care home environment, RTLS could be used to assess whether individual residents are more sedentary alone in their bedrooms or when mixing with other residents in communal areas. Depending on the findings, some residents may then be best suited to an individual intervention focusing on bedroom-based sedentary behavior while other residents may be more suited to a group intervention focusing on communal area sedentary behavior.

### Wearable Cameras

**Figure 4:**
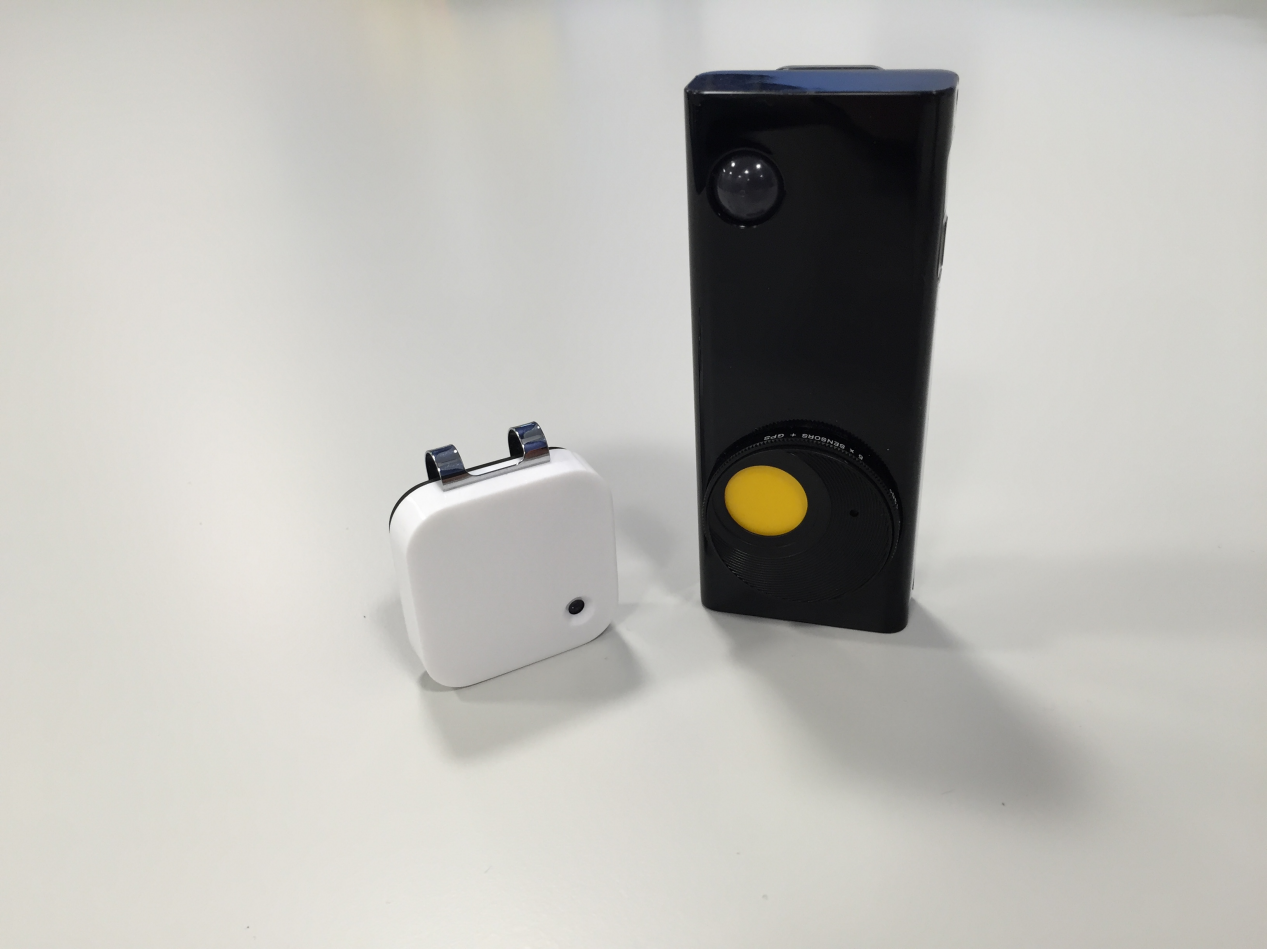
Hardware of two wearable cameras. Shown on the left is the Narrative clip [201] and on the right the Autographer [200].

Recent interest has accumulated in the use of wearable cameras in physical activity and sedentary behavior research, mirroring the growth of the life-logging and quantified-self communities. However, several of the wearable cameras identified in this review appear to have limited public health utility due to very short (eg, 1.5 hours) of battery life. The most popular wearable camera in a research setting is the Microsoft SenseCam. Worn on a lanyard around the neck and containing sensors such as passive infrared, accelerometer, and gyroscope, this device automatically captures a first-person picture at a frequency of approximately 20 seconds. The device has a battery life of approximately 16 hours with sufficient memory capacity to store approximately 32,000 images [294]. From initial small-scale pilot studies, it appears that images generated from wearable cameras are a feasible means of assessing active travel behavior [144,294]. Wearable cameras, therefore, provide broader contextual information; however, they can also be used to infer location. Commercially available wearable cameras, such as the Autographer, also provide GPS coordinates alongside the photograph. Two of the most popular wearable cameras are shown in Figure 4.

Unlike pure location measurement technologies such as GPS and RTLS, wearable cameras are able to provide broader contextual information based on the generated images. For example, a succession of images may show a television set. From this, it could be identified that the participant is watching television. Likewise, a succession of images may show a group of people of a similar age to the participant which researchers may be able to classify as time spent with friends; this is important as an individual’s friends may play a role in shaping physical activity behaviors [295].

Despite the encouragement offered by these initial studies, significant ethical, privacy, and analytical issues remain. There is a possibility that participants may be wearing the device during situations in which they do not wish to be photographed. To overcome this, the device allows the user to turn off the device for several minutes should they require privacy. There is also the possibility that the device may take pictures of an individual that participants encounter who does not wish to be photographed. Linked to this is the possibility that individuals may be wearing the device in situations that are unsuitable for photography, such as dropping off or picking up children from school. In an effort to overcome some of these issues [296], an ethical framework has been proposed for the use of wearable cameras in research. The framework includes the issues of informed written consent from participants, privacy and confidentiality, nonmaleficence, and the autonomy of third parties [296].

Alongside these privacy issues is the issue of data analysis. Current data analysis methods are laborious, involving the manual trawling and coding of images. For long-term monitoring this may prove to be prohibitive in the adoption of wearable cameras. Pattern recognition algorithms to semiautomate this process are available from computer scientists; however, there is a need for these to be integrated into device software in a manner which is suitable for end users. Despite these issues, wearable cameras can be used to assess where behavior occurs both indoors and outdoors and may, therefore, be able to supplement GPS to provide a greater range of contextual information.

The preceding discussion of GPS, RTLS, and wearable cameras highlights the principles, limitations, and use in physical activity and sedentary behavior research of each of these three technologies. GPS is the dominant technology used within research to date to assess where physical activity and sedentary time occur. However, the development of RTLS and wearable cameras offers the possibility to incorporate these technologies alongside GPS and accelerometry to provide a more comprehensive behavioral profile which fully elucidates the context, intensity, and duration of the behavior. The present systematic review also identified several other location monitoring technologies, such as RFID and IC tags, that are less "ready to use" than the three main technologies discussed. While these technologies, particularly RFID, may have a substantial research base behind them, there appears to be no "off the shelf" complete system which is readily purchasable for location tracking.

The ability to assess where behavior occurs in an indoor environment may be particularly elucidating for sedentary time. With the ability to assess where sedentary behavior occurs at work (eg, in a meeting room or at a desk) and at home (eg, sofa, desk, or dining table), behavioral researchers would possess a more comprehensive profile of the context in which sedentary behavior occurs, which could further illuminate the most common modes of sedentary behavior.

It is also worth briefly considering available technologies which were not included in this systematic review, largely due to a lack of wearability. Bluetooth low energy (BLE) proximity systems have recently gained in popularity in certain applications. Many of these systems are primarily aimed toward retail applications for the purpose of proximity marketing. In this scenario, small BLE beacons are placed around a retail environment. The customer, as they are perusing the store with a BLE-enabled device such as a mobile phone, then receives targeted marketing and discount offers to their phone based on their proximity to the beacons. For example, when the customer is perusing the carbonated drinks aisle in a supermarket, an offer may be sent to their phone for a particular brand of drink. These systems offer the potential to install BLE beacons within an indoor environment and determine location based on proximity to the beacons.

Of particular note is the recent miniaturization of BLE beacons to the size of a sticker, so suitably small that it may unobtrusively be attached to items such as chairs, bicycles, and sports equipment. This novel "nearables" equipment offers the potential to assess the location and type of behavior.

### Conclusions

This systematic review sought to identify tools which have been used or could be used to asses where physical activity and sedentary time occur. We identified 188 research papers, of which 119 used GPS and 23 used wearable cameras. A total of 76 location tracking devices or systems were used. Systematic Internet search engine searches found 21 wearable camera models, 78 RTLS tags, and 81 GPS devices. This gave a cumulative total of 263 location tracking devices or systems. GPS is the dominant form of location tracking used within physical activity research to date. While GPS is a valid measure of outdoor location, it is unable to be used within an indoor environment.

Recent developments in wearable cameras and RTLS systems have ensured that tools are now available which offer the potential to assess where physical activity and sedentary behaviors occur indoors. Thus, these tools can provide further contextual information, alongside GPS, when used in conjunction with measures of physical activity and sedentary behavior such as accelerometers. Issues and limitations of each technology were identified, including privacy, data analysis and interpretation, and common data processing methodologies. The integration of accelerometry, GPS, and a technology capable of assessing indoor location would provide researchers with the ability to assess the indoor and outdoor location of physical activity and sedentary behavior. Future research should, therefore, investigate the feasibility of incorporating these technologies, with particular reference to the wearability of the devices, the integration of data streams, and the generation of meaningful behavioral outcomes.

## Appendix

Multimedia Appendix 1 Full version of Table 2.

Multimedia Appendix 2 Full version of Table 5.

Multimedia Appendix 3 Full version of Table 6.

## Abbreviations

AOA: angle of arrival
BL: battery life
BLE: Bluetooth low energy
CCTV: closed-circuit television
CR: camera resolution
Dim: dimensions
EMA: ecological momentary assessment
FPS: frames per second
GIS: geographic information system
GPS: global positioning system
IC: integrated circuit
IEEE: Institute of Electrical and Electronics Engineers
I/O: indoor/outdoor
IR: infrared
Man: manufacturer
MET: metabolic equivalent
MVPA: moderate-to-vigorous physical activity
N/A: not applicable
NIHR CLAHRC for EM: National Institute for Health Research Collaboration for Leadership in Applied Health Research and Care—East Midlands
PALMS: Personal Activity and Location Measurement System
Refs: references
RF: radio frequency
RFID: radio-frequency identification
RSSI: received signal strength indicator
RTLS: real-time locating system
SF: sampling frequency
TDOA: time difference of arrival
TOA: time of arrival
TOF: time of flight
TWR: two-way ranging
UWB: ultra wide band
