# Supplementary material for: Technologies That Assess the Location of Physical Activity and Sedentary Behavior: A Systematic Review
Source: J Med Internet Res. 2015 Aug 5;17(8):e192. doi: 10.2196/jmir.4761 (PMC4705371; doi:10.2196/jmir.4761)
Supplement: Multimedia Appendix 1 [file jmir_v17i8e192_app1.pdf]

Table 2. Summary of Global positioning systems used to date in published research

Manufacturer	Model	Battery life	Dimensions	Weight	Wear site	Cold start time	Storage capacity	References and notes
**Garmin**	Foretrex 201	15 hours	8.4cm x 4.3 cm x 1.8cm	78g	wrist	45 seconds	10,000 points	[16,42-53]
**Garmin**	Forerunner 305	Typically 10 hours	5.3cm x 6.8cm x 1.7 cm	77g	Wrist	45 seconds		[54-57]
**Garmin**	Etrex	22 hours			Neck and thigh			[58,59]
**Garmin**	Forerunner 201				Wrist	Change in direction or speed resulting in 3-55 seconds		[60,61]
**Garmin**	Foretrex 101	12 Hours			Wrist	10 seconds		[62,63]
**Garmin**	Forerunner 205	10 hours	53 x 69 x 18mm	77g	Wrist	45 seconds	72,00 points	[47,52,64-67]
**Garmin**	60				Pocket of backp	0.5 Hz		[68,69]

					ack			
**Garmin**	12CX				Harness	2 seconds		[70,71]
**Garmin**	Forerunner 305				Wrist	1 Hz		[57,72]
**Garmin**	Forerunner 110							[73]
**Telespial systems**	Trackstick II	16-36 hours in full power, 2days-1 week in power save	11.4cm x 3.1cm x 1.9cm			Maximum of 52 seconds	1mb of flash memory	[74,75]
**Global sat**	DG100	20-24 hours			Waist	5, 15 or 30 seconds	Up to 50,000 data points	[47,69,76-81]
**GPSports**	SPI-Elite				On back via a harness	1 Hz		[82-91]
**GPSports**	WI SPI				In harness on back	1 Hz		[87,92]

Table 2. Summary of Global positioning systems used to date in published research

**GPSports**	SPI-PRO				In harness on back	5Hz		[93-96]
**GPSports**	SPI-10				In harness on back	1Hz		[87,88,95,97-99]
**Catapult innovations**	MinimaxX	5 hours	8.8 cm x 5 cm x 1.9 cm	67 g	On back via a harness		1gb flash memory	[84,95,100-109]
**Telespial systems**	Super	4-8 days			Waist	5 or 15 seconds		[110,111]
**Qstarz**	BT1000X	42 hours	72 x 47 x 20mm	65g	Pouch on belt	35 seconds, 5 seconds, 15 seconds	400,000 points	[37,40,52,81,112-126, 296]
**Leica**	System 500				In a rucksack	5Hz		[127,128]
**Geostats**	Geologger				In a rucksack	1 second		[129,130]
**Wintec**	Easy showily	Up to 15 hours			Wrist	5, 15 or 30 seconds		[47]

**Wintec**	WBT-202	28 hours	64 x 40 x 14 mm	55g		34 seconds	260,000 points	[131]
**Globalsat**	BT335	25 hours			Waist	30 seconds		[132-136]
**Globalsat**	TR203	8 hours	79 x 42 x 18mm	70 g		36 seconds	150,000 points	[52]
**IGotU**	GT 600	30 hours	46 x 41 x 14 mm	37g		35 seconds	262,000 points	[52]
**IGotU**	GT120	3 days		<50g	Lanyard around neck		3 days	[137]
**FRWD**	B100	12 hours	95 x 55 x 15 mm	85g		42 seconds		[52]
**Starsnav**	BTS-110	22 hours	76 x 46 x 20mm	57g		42 seconds	250,000 points	[52]
**Adeo**	GPS fitness trainer				Right arm			[72]
**Polar**	RS 800 G3 Heart rate				Wrist			[72]

Table 2. Summary of Global positioning systems used to date in published research

	monitor							
**Mobitest GSL**		Up to 100 hours					128-512 Mb	[138]
**Transystem inc**	GPS logger 747A	32 hours	46.5 x 72.2 x 20 mm	64g	Hand bag or trouser pocket	35 seconds	125,000 points	[139]
**Sparkfun electronics**	Kinetamap	Approximately 20 hours			Waist	42 seconds		[140]
**Miscellaneous systems including prototypes, mobile phone based systems and receivers**								[57,141-155]
