# Supplementary material for: Technologies That Assess the Location of Physical Activity and Sedentary Behavior: A Systematic Review
Source: J Med Internet Res. 2015 Aug 5;17(8):e192. doi: 10.2196/jmir.4761 (PMC4705371; doi:10.2196/jmir.4761)
Supplement: Multimedia Appendix 2 [file jmir_v17i8e192_app2.pdf]

Table 5. Summary of commercially available real-time locating systems unused in research to date

Manufacturer	Model	Infrastructure/ method	Dimensions	Accuracy
**Ekahau [214]**	A4	Wi-fi, RSSI and triangulation	45 x 55 x 19mm	1m
	A4+	Wi-fi, RSSI and triangulation	45 x 55 x 19mm	1m
	B4	Wi-fi, RSSI and triangulation	60 x 90 x 8.5mm	1m
	W4	Wi-fi, RSSI and triangulation	51.5 x 50 x 17.5mm	1m
**Ubisense [215]**	Series 7000 industrial	UWB, TOA, AOA	71 x 64 x 47 mm	15cm
	Series 7000 compact	UWB, TOA, AOA	38 x 39 x 16.5mm	15cm
	Series 700 tool tags (integrated unit)	UWB, TOA, AOA	107 x 39 x 30mm	15cm
	Series 7000 slim tag	UWB, TOA, AOA	83 x 42 x 11mm	15cm
	Series 700 intrinsically safe tag	UWB, TOA, AOA	38 x 39 x 25.5 mm	15cm
	Series 9000 compact tag	UWB, TOA, AOA	38 x 39 x 16.5mm	15cm
	Series 7000 Trimode tag	UWB, TOA, AOA	71 x 64 x 47mm	
**Aeroscout [216]**	T2 tags	Wi-fi, RSSI, TDOA	62 x 40 x 17mm	
	T2s	Wi-fi, RSSI, TDOA	45 x 31 x 18mm	

	T2-EB	Wi-fi, RSSI, TDOA	85 x 59 x 19mm	
	T3	Wi-fi, RSSI, TDOA	74 x 50 x 10 mm	
	T4b	Wi-fi, RSSI, TDOA	69 x 48 x 21mm	
	T4P	Wi-fi, RRSI, RDOA	180 x 85 x 45 mm	
	T5a	Wi-fi, RSSI, TDOA	62 x 40 x 17mm	
	T5b	Wi-fi, RSSI, TDOA	113 x 59 x 19mm	
	T5c	Wi-fi, RSSI, TDOA	113 x 59 x 19mm	
	T5h	Wi-fi, RSSI, TDOA	62 x 40 x 17mm	
	T6	Wi-fi, GPS, RSSI, TDOA	100 x 80 x 55 mm	
**Zebra [217]**	Where tag IV	Wi-fi, TDOA	43.7 x 66 x 21.3 mm	2 metres
	Wheretag III	Wi-fi, TDOA	21 x 66 x 44 mm	
**Elpas [218]**	Asset tracking tag (healthcare)	RF, IR, LF		Sub room
	Healthcare positioning tag	RF, IR, LF		Sub room
	High risk security bracelet (healthcare)	RF, IR, LF		Sub room
	Infant protection bracelet	RF, IR, LF		Sub room

	Personal safety bracelet	RF, IR, LF	Sub room
	Personnel identity badge (Healthcare)	RF, IR, LF	Sub room
	Asset tracking tag (commercial)	RF, IR, LF	Sub room
	Lone worker transmitter	RF, IR, LF	Sub room
	Low profile asset tag	RF, IR, LF	Sub room
	Personnel identity badge (commercial)	RF, IR, LF	Sub room
**Centrak [219]**	Asset tags	Wi-fi	
	Staff tags	Wi-fi	
	Patient tags	Wi-fi	
**Teletracking [220]**	Whole system	IR, RSSI	Bed/bay
**Sonitor [221]**	Whole system	Wi-fi, ultrasound, RSSI	I feet
**Versustech [222]**	Clearview badge	IR,	Up to chair level
**Radianse [223]**	T-100	RF, IR	Up to bed level
	T-400	RF, IR	Up to bed level
	T-600	RF, IR	Up to bed level
**Securecare [224]**	EnvisionIT	Wi-fi	30 cm
**Mojix [225]**	E-Location	Passive RFID	Within 1 metre

**Assetworks [226]**	Whole system	Tag to tag RFID		
**Tempsys [227]**	Fetch system	RF and ultrasound, TDOA		½ metre
**Awarepoint [228]**	Asset tags	Zigbee,	1.8 x 1.3 x 0.5 inch	Up to bay level
	Wearable tag	Zigbee,	1.8 x 1.3 x 0.5 inch	
**Comita [229]**	Whole system	WI-FI		
**Trackit [230]**	Asset and patient tag	UWB, TDOA		< 1 feet
**Nebusens [231]**	Sirius Quantum	Zigbee,	22 x 32.72 x 5 mm	1 metre
**Essensium [232]**	Mobile nodes	Wide over narrowband RF, TWR, TOF	19.8 x 8.8 cm	typically 50cm
**Pluslocation [233]**	R1 badge tag	UWB, TDOA	38 x 78 x 9.6mm	< 1m
	R1 asset tag	UWB, TDOA	13 x 36 x 33mm	<1m
	R2 tags	UWB, TDOA	87 x 42 x 10	<1m
**Technical life care [234]**				
**Airista [235]**	TDOA 1P66 tag	Wi-fi, RFID, RSSI, TDOA	180 x 90 x 40mm	1-2m
	TDOA tag	Wi-fi, RFID,	53 x 35 x	1-2m

		RSSI, TDOA	15mm	
	AUTP-W tag	Wi-fi, RFID, RSSI, TDOA	70 x 44 x 16	
	AUTW-W tag	Wi-fi, RFID, RSSI, TDOA	68 x 42 x 18mm	
	ATP-W	Wi-fi, RFID, RSSI, TDOA	86 x 54 x 8mm	
	ATA-W tag	Wi-fi, RFID, RSSI, TDOA	53 x 38 x 16mm	
**Conduco [236]**				
**Luminosity [237]**		IR, RF		
**Purelink [238]**	Personnel tracking tag	RFID	85 x 54 x 4mm	2 metres
	Equipment tracking tag	RFID	85 x 54 x 4.5mm	2 metres
**Sanitag [239]**	Staff tag	RF, RSSI, TOF	90 x 61 x 5 mm	2.5 metres
	Patient tag	RF, RSSI, TOF	43 x 36 x 10 mm	2.5 metres
**Aidarfid [240]**		Wi-fi, RF		
**Openrtls [241]**	tag	UWB, TDOA, TWR	66 x 44 x 17 mm	10cm
**Bespoon [242]**		UWB, TWR, triangulation		
**Ecived [243]**	Loulan	RFID, ultrasonic		5 cm

**Skytron [244]**		Wi-fi,		Up to chair level
**Logi-tag [245]**		RFID		
**Red point positioning [246]**	Tag	UWB,	56 x 32 x 14	< 0.5 metres
**Bordatech [247]**	Wrist tag	RFID,		Sub room
**Point RF [248]**	Dynamic positioning system	RF, IR, LF		1.5 metres
