# Supplementary material for: Technologies That Assess the Location of Physical Activity and Sedentary Behavior: A Systematic Review
Source: J Med Internet Res. 2015 Aug 5;17(8):e192. doi: 10.2196/jmir.4761 (PMC4705371; doi:10.2196/jmir.4761)
Supplement: Multimedia Appendix 3 [file jmir_v17i8e192_app3.pdf]

Table 6. Summary of commercially available global positioning systems unused in research to date

Manufacturer	Model	Battery life of wearable component	Dimensions
**Trackstick [249]**	Trackstick mini	3-14 days	3 1/2 x 1 1/2 x 3/8 inch
	Trackstick II	16h-2 days (AAA)	4 1/2 x 1 1/4 x 3/4 inch
	Super trackstick	3 days-3 weeks (AAA)	4 1/2 x 1 1/4 x 3/4 inch
**Trackershop-UK [250]**	Pro-pod5	14-15 days	6.35 x 4 x 2.5 cm
	Pro pod 4	8-11 days	6.25 x 4 x 2 cm
	The chameleon	24 hours	60 x 23 x 12mm
	Personal GPS tracker		77 x 47 x 20
**Gotek 7 [251]**	Prime 1.0	10 days normal. Up to 12 months with 1 update per day	
	Prime 2.0	15 days normal up to 14 months (1 per day)	65 x 42 x 25mm
**Carewhere [252]**		5-7 days	
**Pocketfinder [253]**		Up to one week	
**BluetrackGPS trackers [254]**	Prime lite	100-170h (5min) 150-220h (10min)	67.8 x 37 x 20 mm
	Prime 1300	10 days in normal mode	

	Prime 2000	15 days in normal mode	65 x 42 x 25mm
	Bond 2000	10-15 days normal use	62 x 34 x 31 mm
	Bond 5800	20-40 days normal use	70 x 40 x 44 mm
	Bond 11600	40- 60 days normal use	140 x 35 x 33 mm
	Bond 17400	80 -100 days normal use	200 x 35 x 30 mm
	The sniper	2 months live mode (2-4 months in battery save)	60 x 10 x 45 mm
	GPS belt		Depends on waist size
	Slim jim	4- 6 days normal use	115 x 35 x 5 mm
**Trackinapack [255]**	Advanced	Up to 10 days	2.63 x 1.38 x 0.79 inches
	Advanced plus	Up to 15 days	2.5 x 1.5 x 0.79 inches
**Protect my kids [256]**		7 days	
**Amber alter GPS [257]**		Up to 40 hours	2.8 x 1.5 x 0.8 inch
**Traclogik [258]**	Guardian GPS	100-220 hours	67.8 x 37 x 20 mm
	Guardian pro GPS	2-14 days	62.5 x 40 x 25mm
	Covert 2000	10-15 days	61 x 34 x 31
**Laipac [259]**	s911 lola	up to 5 days in sleep mode	5.4 x 4 x 1.6

			cm
	s911 bracelet		5 x 4.4 x 1.5cm
	s911 personal locator		100 x 45 x 25mm
**Loc8tor [260]**		Up to 9 months in power save. 3-14 days normally	68 x 36 x 20mm
**Meitrack [261]**	MT90	14 hours	77 x 47 x 20mm
**SonikGPS [262]**	SNK001		
**Global tracking group [263]**	UBI-5000E	Up to 30 days	67.5 x 40 x 21mm
**GPS intergrated [264]**	PGT2		92 x 44 x 18mm
	PGT3		92 x 44 x 18mm
**Buddi [265]**			
**Key tracker [266]**	Personal tracker		22 x 58 x 38mm
**RM tracking [267]**		up to 6 days at 2 hours per day	3.9 x 2.3 x 0.9 inch
**Landairsea [268]**	Silvercloud realtime GPS tracker	5-6 days at 2 hours per day	3.9 x 2.26 x 0.9 inch
	Tracking key pro	2 week (4h), 4 week (2h), 6 week (1h per day)	3.01 x 1.95 x 1.4 inch
**Dynaspy [269]**	World tracker enduro pro	Up to 150h	64.66 x 43.19 x 27.7mm

	Ultra accurate real time	Up to 150h	64.66 x 43.19 x 27.7mm
**Whereible GPS [270]**	Wheritrack		3 1/8 x 1 9/16 x 3/8 inch
**Ilotech [271]**	Triloc	60+ hours	52 x 69.5 x 17 mm
**GTX corp [272]**	Prime AT	up to 16 days	67 x 37 x 20 mm
	GT200	50-60h (5min), 70-80h (10min), 120-150h (sleep)	74.8 x 42.8 x 17.5 mm
	Smart sole	2-3 days	Depends on show size
	VL 2000	54-108 hours	72.2 x 38.4 x 18.7 mm
**Biosensics [273]**	PAMsys		
**Reconinstruments [274]**	Recon jet	4 hours	
**Nike [275]**	Sportwatch GPS	8h with average use	1.5 x 10.1 x 0.6 inch
**Garmin [276]**	Forerunner 620	6 weeks (watch) 10 hours (training)	45 x 45 x 12.5 mm
	Forerunner 220	6 weeks (watch) 10 hours (training)	45 x 45 x 12.5 mm
	Forerunner 910XT	up to 20 hours	54 x 61 x 16 mm
	Forerunner 920XT	24 hours	48 x 55 x 12.7 mm
	Forerunner 610	4 weeks (watch), 8h (training)	45.7 x 63.5 x 14.2cm

	Forerunner 310XT	up to 20 h	54 x 56 x 19cm
	Forerunner 210	3 weeks (watch), 8 hours (training)	45 x 69 x 14 mm
	Forerunner 110	3 weeks (power save), 8h (training)	4.5 x 6.9 x 1.4cm
	Forerunner 10	5 weeks (watch), 5 h (training)	45.5 x 57.2 x 15.7 mm
	Vivoactive	10 hours (up to 3 weeks in smart watch mode)	43.8 x 38.5 x 8 mm
	Fenix 3 sapphire	Up to 20 hours (6 weeks in watch mode)	51 x 51 x 16 mm
	Fenix 3	Up to 20 hours (6 weeks in watch mode)	51 x 51 x 16 mm
	Epix	24 hours (16 weeks in watch mode)	50.8 x 53.3 x 17.8 mm
	Forerunner 15	8 hours (5 weeks in watch mode)	45.5 x 57.2 x 15.7 mm
	Tactix	50 hours (5 weeks in watch mode)	49 x 49 x 17 mm
	Fenix 2	20 hours (5 weeks in watch mode)	49 x 49 x 17 mm
**Revolutionary tracker [277]**	RT-01		
	RT-02		
**[278] Everon**	Vega GPS bracelet		
**Trax family [279]**	Trax	1 day	38 x 55 x 10 mm
**Ninja tracking systems [280]**	Ninja tracker	300-400 hours standby time, 5 minute reporting time, 100 – 170 hours, 10 minute reporting:150-	

		220 hours	
**Personal GPS trackers [281]**	Personal GPS tracker	Up to 7 days	65 x 40 x 18 mm
	GPS tracker watch	24-48 hours	60 x 45 x 18 mm
	Mini GPS tracker	2-4 days	58 x 22 x 11 mm
**Retrievor [282]**			28 x 15 mm
**Duotraq [283]**	DQ 300	140 hours	68.5 x 38.5 x 23.5 mm
**Mind me [284]**	Mind me locate	48 hours	65 x 35 x 17 mm
**Bubble tracker [285]**	Personal GPS tracker		79 x 42 x 18 mm
